# Efficacy of genogroup 1 based porcine epidemic diarrhea live vaccine against genogroup 2 field strain in Japan

**DOI:** 10.1186/s12985-018-0940-8

**Published:** 2018-02-02

**Authors:** Tetsuo Sato, Kazuki Oroku, Yoshiyuki Ohshima, Yoshiaki Furuya, Chihiro Sasakawa

**Affiliations:** Nippon Institute for Biological Science, 9-2221-1 Shin-machi, Ome, Tokyo 198-0024 Japan

**Keywords:** Lactogenic immunity, Porcine epidemic diarrhea, Porcine epidemic diarrhea virus, Vaccine

## Abstract

**Background:**

Porcine epidemic diarrhea (PED) is a lethal infectious disease in suckling piglets with symptoms including watery diarrhea caused by PED virus (PEDV). Since the late 1990’s, live vaccines based on genogroup 1 virus have been used in Japan, and a significant amount of the vaccine has been used even after new genogroups invaded in 2013. In this study, we evaluated the effect of a conventional PED live vaccine on a newly prevalent genogroup 2 field strain in experimental and field situations.

**Methods:**

Two pregnant sows were administered twice the live vaccine before farrowing. A pregnant sow was served as a negative control. All newborn piglets were challenged with the genogroup 2 virus, and clinical signs were monitored for 7 days post challenge. PEDV-specific immune responses in serum and milk of the sows were assayed by virus neutralization assay. The efficacy of PED live vaccine in vaccinated or non-vaccinated farms was evaluated by comparing the mortality rate of suckling piglets after the onset of PED.

**Results:**

The challenged piglets exhibited watery diarrhea with or without vaccination. However, the clinical score of piglets born from vaccinated sows significantly improved after the 4th day of the challenge. The survival rate of piglets in the vaccinated group at the end of the experimental period was 80%, whereas in the control group was 0%. Neutralizing antibody titers in serum and milk of control sow was negative throughout the experimental period, whereas high titers were observed in the vaccinated sows. The vaccinated farms significantly reduced the mortality rate of suckling piglets after the onset of PED, compared to farms not vaccinated.

**Conclusions:**

The conventional PED live vaccine induced the lactogenic immunity to vaccinated sows and showed partial protection against the genogroup 2 virus both under the experimental and field conditions.

**Electronic supplementary material:**

The online version of this article (10.1186/s12985-018-0940-8) contains supplementary material, which is available to authorized users.

## Background

Porcine epidemic diarrhea (PED) is a viral infection caused by oral infection with PED virus (PEDV) and causes symptoms including watery diarrhea and anorexia [1]. The mortality rate of PED is as high as 50 to 100% in piglets up to 1 to 2-week-old. Weaning pigs may have transient diarrhea or remain asymptomatic even if they have been orally administrated the virus.

PED was firstly identified in early 1980’s in Japan, and a nationwide pandemic was also recorded in 1996. In September 2013, PED was reported in Okinawa Prefecture after an interval of 7 years [2], the virus spread rapidly nationwide. The isolated viral strain was divided into previously reported strains in Japan and genetically similar to that reported in the US in May 2013. It was belonged to genogroup 2 according to the phylogenetic analysis of viral whole-genome sequences, also called the US prototype-like strains [2]. Subsequently, another genogroup 2 field strain called the INDEL (which has the insertion and deletion in the spike gene) strain was also isolated in Japan [3]. Further, Tottori2 strain with a larger deletion in the spike gene was reported in October 2014 [4]. Currently, the several genogroup 2 strains are circulating in Japanese pig farms as in another outbreak countries.

Vaccination is an ideal control measure to prevent infectious diseases [5]. Since 1990’s, intramuscular live or inactivated vaccines were introduced in Asian countries [6, 7]. Today, an oral vaccine was commercialized in South Korea and the Philippines [8]. All these vaccines are based on genogroup 1 strains and are different from the recent global epidemic strains of PEDV. Since PED outbreaks in North America in April 2013, the vaccine based on a truncated PEDV spike gene produced in the SirraVax^SM^ RNA Particle Technology platform, and an inactivated whole virus vaccine formulated with an adjuvant were used in the US from 2013 to 2014, respectively [9, 10]. These North American vaccines are based on genogroup 2 strains. Over 6 million doses of the live vaccine produced by genogroup 1 strain had been supplied to Japanese domestic market during 2014 and 2015. Although numerous vaccines have been utilized in the pig farms, PED outbreaks have been still reported on vaccinated farms in Japan.

In the present study, to evaluate the effect of the conventional live vaccine based on the genogroup 1 strain against the emerging field strain, we isolated a genogroup 2 field strain and performed a challenge test using PEDV antibody- and PEDV free pregnant sows. In addition, we compared the mortality rate of suckling piglets among PED affected farms to assess the effect of the live vaccine in the field situation.

## Methods

### Virus isolation

A genogroup 2 field strain was isolated in Vero cell culture, to be used as inoculum in the challenge test. The Jejunum from six piglets which show watery diarrhea were obtained from a PED affected farm. These specimens were used for generating a 10% (*w*/*v*) homogenate in Eagle’s minimum essential medium (MEM; Life Technologies). The homogenate was centrifuged at 1,500×g for 15 min at 4°C. The supernatant was filtered through a 0.22 μm pore-size filter.

Virus isolation of PEDV was performed on Vero cells as previously described [11]. In brief, Vero cells were cultured in MEM supplemented with 10% heat-inactivated fetal bovine serum, 0.3% tryptose phosphate broth (TPB), and antibiotics. Before inoculation, the culture medium was removed, and the monolayer cells were washed twice with inoculation medium: MEM supplemented with 0.3% TPB, 0.02% yeast extract, and 10 μg/ml trypsin (Sigma). The specimens were inoculated with inoculation media, and after adsorption at 37°C for 90 min, the inoculation medium was removed, and then 4 ml of inoculation medium was added to each well. The cultures were incubated at 37°C and examined for cytopathic effect (CPE) once daily. Virus titration was performed in 24-well plates with 10-fold serial dilutions performed in quadruplicate per dilution. The cultures were examined for CPE once daily, and the virus titers determined according to the Reed Muench method [12] and expressed as the 50% tissue culture infective dose (TCID_50_)/ml.

### Comparison of the spike gene sequence with another PEDV strains

Viral RNA was extracted from virus cultures using Trizol (Life Technologies) according to the manufacturer’s instructions. The cDNA was synthesized from viral RNA using Transcriptor High Fidelity cDNA Synthesis kit (Roche Diagnostics). PCR was performed on cDNA using Phusion® High-Fidelity DNA Polymerase (Finnzymes). The spike gene was amplified with the primers according to a previous report [13]. The nucleotide sequences of spike gene were determined directly from PCR-amplified products by the Fasmac sequencing service. The spike gene sequence of an isolate, designated as MZ0116-2/2013, was deposited in GenBank (accession no. LC258163). DNA alignments and phylogenetic tree analysis were performed by using the maximum-likelihood method with the ClustalW method, the general time reversible nucleotide substitution model and bootstrap tests of 1,000 replicates in MEGA software [14]. The potential N-linked glycosylation sites were predicted by the NetNGlyc 1.0 Server [15].

### Animal immunization and challenge test

Three pregnant sows were confirmed serologically negative for PEDV neutralizing antibody before the first vaccination. The vaccine (Nisseiken Co., Ltd., lot No. B43-1) contains P-5V strain more than 10^4.5^ TCID_50_/2 mL/dose, which is attenuated by the Vero cell culture. P-5V is a vaccine strain belonging to genogroup 1. Two pregnant sows (No. 677 and 826) were vaccinated intramuscularly at 6 and 2 weeks before farrowing with a vaccine dose each time. A non-vaccinated pregnant sow (No. 72) served as the negative control. Serum samples of the sows were collected at the first pre-vaccination, the 4 and 11 days after farrowing to measure the neutralizing antibody titers against PEDV. Also, milk samples were collected at the day of farrowing, 4 and 11 days after farrowing. The collected serum and milk of sows were stored at − 20 °C until the analysis.

All of the newborn piglets (vaccinated group: *n* = 15; control group: *n* = 11) were only fed the milk including the colostrum that has been derived from the vaccinated or unvaccinated sows during the experimental period. No additional feeding of artificial milk to piglets was carried out. All of the piglets were orally administered the 5th-passage virus of MZ0116-2/2013 strain 10^5.0^ TCID_50_/ml at 4 days after farrowing. The clinical signs of piglets and sows were monitored until 7 days post challenge. Clinical signs were recorded as follows, score 0; normal stools, score 1; loose stools, score 2; diarrhea, score 3; watery diarrhea, score 4; death.

### Neutralization test

Virus neutralizing antibody test in serum and milk was carried out according to the previous report [11]. Briefly, virus neutralization test was performed in 24-well plates with serum of two-fold serial dilutions in triplicate per dilution. A hundred μl of diluted serum was mixed with an equal volume of P-5V virus supernatant containing 200 TCID_50_/0.1 ml and then incubated at 37°C for 90 min. The mixture was inoculated to confluent Vero cell cultures grown in each well and incubated at 37°C for 90 min. After adsorption, the inoculum was removed, and the monolayers were washed twice with post-inoculation medium. Five hundred μl of post-inoculation medium was added to each well and incubated 37°C. P-5V strain has been completely adapted to Vero cells, shows an apparent CPE. The neutralizing antibody titer was determined at 1-week post inoculation. The collected milk was centrifuged at 10,000×g for 15 min at 4°C, and then the supernatant was subjected to a neutralization test in the same procedure as serum. The virus neutralizing antibody titer was expressed as the reciprocal of the highest dilution of serum capable of preventing viral replication.

### Comparison of the mortality rate of suckling piglets among PED affected farms

The efficacy of PED live vaccine in vaccinated or non-vaccinated farms was evaluated by comparing the mortality rate of suckling piglets after the onset of PED. Three farrow-to-finish farms were monitored in this study. All the farms had experienced an outbreak of PED for the first time in April 2014, and the situation and measures of the farms listed are summarized in Table 1. In farm A and B, the live vaccine (transmissible gastroenteritis (TGE)/PED combined vaccine, Nisseiken Co., Ltd) had been administered once or twice into pregnant sows according to the manufacturer’s instructions. Pregnant sows were not vaccinated in farm C. Controlled oral exposure of PEDV contaminants (feedback) to pregnant sows were performed in farm A and C after the onset of PED outbreak. The mortality rate of suckling piglets was calculated from the number of dead piglets per the piglets born every week.

**Table 1:** Outline of farms affected by PED

Farm	Production system	Number of sows	Live vaccine inoculation to pregnant pigs	Feedback to pregnant sows
A	Farrow-to-finish	800	Inoculated once or twice during gestational period	Yes
B	Farrow-to-finish	800	Inoculated twice during gestational period	No
C	Farrow-to-finish	943	Not inoculated	Yes

### Statistical analysis

The clinical score, neutralization titer, and mortality rate of suckling piglets in field farms were analyzed with a repeated analysis of variance (ANOVA). If the repeated measures ANOVA showed a significant effect, a one-way ANOVA with pairwise testing using Tukey’s adjustment was performed at each time point.

## Results

### Virus isolation and sequence analysis of spike gene

**Fig. 1:**
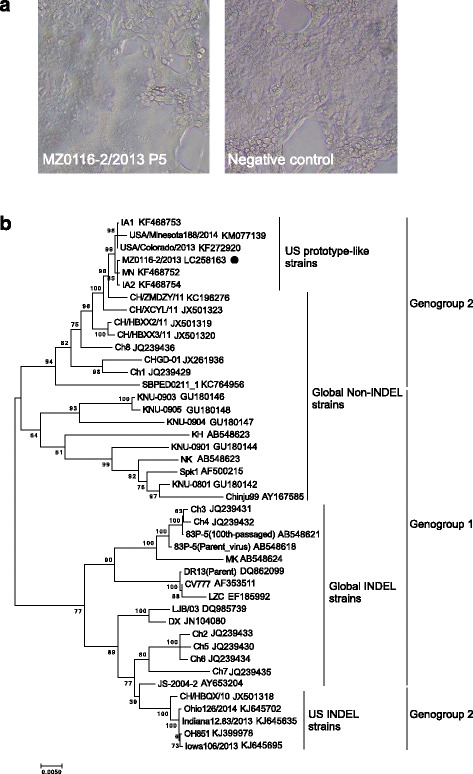
Cell culture and phylogenetic analysis of PEDV MZ0116-2/2013 strain. **a** CPE of MZ0116-2/2013 in Vero cells. Vero cells were infected with PEDV MZ0116-2 P5 isolates. At 48 h post-infection, CPE was recorded. **b** Phylogenetic tree based on the spike gene sequence of PEDV field isolates. Bootstrap values are represented at key nodes. Scale bar indicates nucleotide substitutions per site. The name of the strains and GenBank accession are shown. The filled circle marks MZ0116-2/2013

A PEDV isolate was obtained from the field samples. The isolated virus was designated MZ0116-2/2013. MZ0116-2/2013 exhibited a distinct CPE characterized by cell fusion and syncytium formation as of passage 0. The isolate was further serially passaged in Vero cells for a total of 5 passages (P1 - P5). The isolate had a virus titer of 10^5.5^ TCID_50_/ml at P5. The CPE was observed within 24 h post inoculation during the propagation process (Fig. 1a).

Phylogenetic analysis based on the spike gene sequences of 43 PEDV strains shows distribution between just 2 major branches that have or do not have INDEL. The non-INDEL strains constituted the first branch; the second branch contained INDEL strains. MZ0116-2/2013 strain has been confirmed to be in US prototype-like non-INDEL, genogroup 2 sublineage (Fig. 1b).

**Fig. 2:**
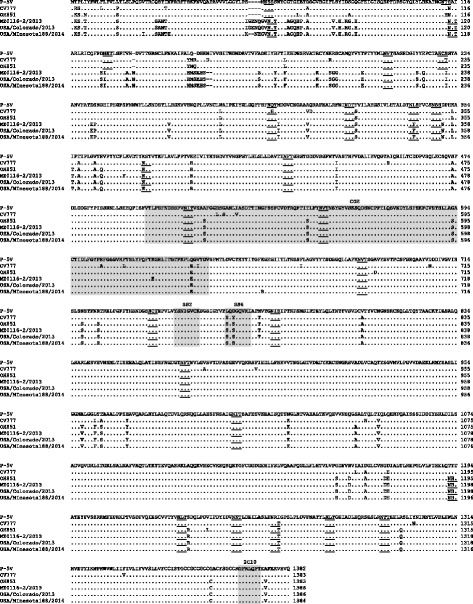
Alignment of the amino acid sequences of the spike protein of PEDV. Dots represent amino acids that are identical to those in the P-5V. Potential N-linked glycosylation sites are underlined. Grey boxes indicate the COE region (498-637) and neutralizing epitopes (747-754; SS2, 763-770; SS6 and 1367-1373; 2C10)

The PEDV spike protein plays important roles in interactions with cellular receptors during virus entry, growth adaptation in vitro, the induction of neutralizing antibodies and alteration of pathogenicity in vivo [16]. As shown in Fig. 2, we compared the amino acid sequences of P-5V vaccine strain with the newly emerging strains. Previous studies of the PEDV spike protein have identified neutralizing COE region (498-637 aa), epitopes SS2 (747-754 aa), SS6 (763-770 aa), and 2C10 (1367-1373 aa) [17–19]. There was no difference in the epitopes SS2 and 2C10 between genogroup 1 and 2 viruses, however, two amino acid differences (Leu_763_ and Asp_765_) were found in the SS6 of P-5V compared to the genogroup 2 viruses. In the P-5V strain, four amino acids (Ala_516_, Thr_548_, Gly_593_, and Gln_632_) were different from all genogroup 2 viruses, and one amino acid (Lys_620_) was different from the MZ0116-2/2013 strain. The Spike protein is also known as a type I glycoprotein [20], and the P-5V vaccine strain contains 22 potential N-linked glycosylation sites. The predicted N-linked glycosylation sites were very similar among genogroup 1 and 2 viruses, however, there were three different N-linked glycosylation sites between the P-5V vaccine strain and the US prototype-like strains (position at Asn_127_, Asn_229_, and Thr_1192_).

### Clinical symptoms in challenge test

**Fig. 3:**
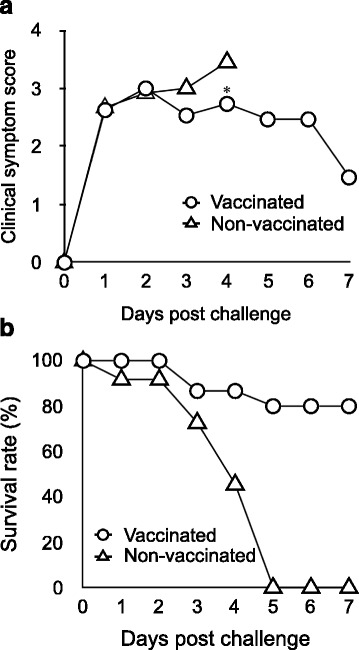
Clinical symptom score and survival rate in the challenged piglets. **a** Mean clinical symptom score of suckling piglets. Clinical symptom score: 0 = Normal stool, 1 = Loose stool, 2 = Diarrhea, 3 = Watery diarrhea, and 4 = Death. **b** Survival rate of the challenged piglets. * means significantly different results as *p* < 0.05

In this study, the piglets derived from vaccinated or non-vaccinated sows with PED live vaccine were challenged with the MZ0116-2/2013 strain. All of the piglets were observed no clinical signs before the challenge of MZ0116-2/2013 strain (Data not shown). After the challenge, both piglets derived from vaccinated and non-vaccinated sows showed watery diarrhea from Day 1 post-challenge (Fig. 3a). Piglets in the control group showed a marked reduction in survival rates on Day 4 post-challenge and all of them died by Day 5. In contrast, clinical symptoms were relieved after reaching a peak on Day 2 in the piglets suckling from the vaccinated sows. The clinical score of the vaccinated group was significantly lower than that of the control group on Day 4. The survival rate on Day 7 was 0% (0/11) in the control group and 80% (12/15) in the vaccinated group (Fig 3b). The vaccinated sows showed anorexia transiently, whereas feces were normal during the experimental period. The non-vaccinated sow showed anorexia and diarrhea on the day after the challenge or later. The milk secreted from the non-vaccinated sow was reduced, and no milk could be collected by human hand on Day 2 post-challenge or later. Therefore, the piglets derived from non-vaccinated sow were observed to drink a lot of water instead of taking less milk. However, undigested milk was filled in the stomach of all piglets that died during the experimental period.

### Neutralizing antibody titer in serum and milk of sows

The neutralizing antibody titer in the serum from the two vaccinated sows was 1:32 and 1:128 at the time of the challenge, respectively (Table 2). It was increased to 1:256 and 1:512 on Day 7 post-challenge. The neutralizing antibody titer in the colostrum was 1:64 and 1:128, but their value was below the detection limit on the day of the challenge, and then increased to 1:128 in both sows on Day 7 post-challenge. The non-vaccinated sow remained antibody-negative throughout the test period.

**Table 2:** Neutralizing antibody titer in serum and milk of sows

Sample	Group	Sow number	Colostrum at farrowing	Number of days after challenge		
				0	2	7
Serum	Vaccinated group	826		32	N. T.	256
		677		128	N. T.	512
	Non-vaccinated group	72		< 2	N. T.	< 2
Milk	Vaccinated group	826	64	< 2	2	128
		677	128	< 2	16	128
	Non-vaccinated group	72	< 2	< 2	N. T.	N. T.

*N. T.* Not tested

### Effect of live vaccine in farms affected by PED

**Fig. 4:**
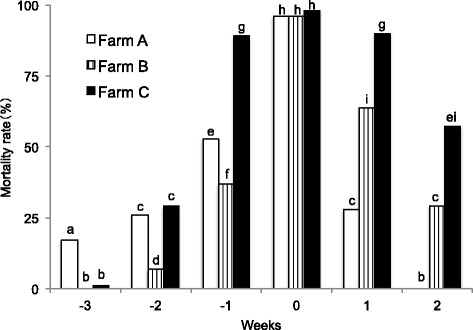
The mortality rate of suckling piglets by age in weeks at field farms with PED. Weeks are indicated as follows: 0 = the mortality rate of piglets born in the week of PED outbreak, − 1, − 2 or − 3 = the mortality rate of piglets in 1, 2, or 3 weeks of age at the week of PED outbreak, respectively, 1 or 2 = the mortality rate of piglets born in the next weeks of PED outbreak. Different letters indicate significant differences among farms (*P* < 0.01)

All the farms recorded a mortality rate of piglets of almost 100% in the week of the onset of PED irrespective of the presence or absence of the vaccination to pregnant sows (Fig. 4. Detailed information about the number of dead piglets is shown in Additional file 1: Table S1). The mortality rate of piglets born in 1 week before and after the onset of PED was reduced in Farm A, where sows were inoculated only once due to limited vaccine supply, as compared to Farm C, where sows were not vaccinated but treated only with feedback. Further, no death was recorded for piglets born 2 weeks after the onset of PED from sows vaccinated twice as directed in Farm A. Similarly, the mortality rate of piglets was reduced during 1 week before and after the week of the onset of PED in Farm B, where sows were treated only with vaccination without feedback, as compared to Farm C, where sows were treated only with feedback.

## Discussion

In this study, we isolated a newly genogroup 2 virus and evaluated the efficacy of a conventional live vaccine (based on genogroup 1 virus). The vaccinated sows showed PED-specific antibody responses, and the neutralizing antibody was secreted in the colostrum. The vaccinated sows also showed a quick antibody response after the challenge. This quick antibody response suggests a possible booster effect by the infection of the sows with the virus shed from piglets. The above outcomes can contribute to reducing clinical signs and mortality rate in piglets derived from vaccinated sows by the live vaccine. Furthermore, the mortality rate before and after the onset of PED was reduced in vaccinated farms compared to a non-vaccinated farm. These results confirm that the PED live vaccine alleviates the damage is due to genogroup 2 field strains in not only experimental condition and also field farms. Further, it was also suggested that sows inoculated with the live vaccine prevented the progression of PED to a more severe condition. Differences in the amino acid sequences and N-linked glycosylation sites found in this study may have an impact on the antigenicity between the vaccine strain and the genogroup 2 strain. It is necessary to carry out future research to elucidate neutralizing cross-reactivity between genogroup 1 and 2 viruses.

PED monovalent and polyvalent (against PED and TGE) live vaccines have been used to prevent PED in Japan since late 1996. These vaccines are intramuscularly injected twice at intervals of 2 or 4 to 8 weeks in pregnant sows during the gestational period. Vaccinated sows secrete milk containing a neutralizing antibody against PEDV after farrowing. To secrete many neutralizing antibodies into the milk of vaccinated sows, it is preferable that the second vaccination is better as close as possible to the farrowing date, it is defined as 2 weeks before farrowing according to the manufacturer’s instructions. The neutralizing antibody taken by piglets into the gastrointestinal tract neutralizes orally infecting PEDV and blocks viral entry into mucosal epithelial cells to relieve PED symptoms. This immunization mechanism of the PED vaccine in piglets is called “lactogenic immunity” and works based on a different action mechanism from the immunization of piglets by maternal antibody in the colostrum. Future study should examine whether the amount of virus is decreased in piglets by lactogenic immunity.

The neutralizing antibody is considered to need to be continuously supplied to the gastrointestinal tract of piglets to prevent the onset of PED through milk. Piglets are infected with PEDV and become more severe when they are unable to take sufficient milk during the suckling period for some reason. Another problem is that when sows with low neutralizing antibody titer are exposed to a large amount of the virus, they develop systemic symptoms associated with reduced milk secretion, limiting the full effect of the vaccine. Considering the above mechanism of the PED vaccine, sufficiently increasing the level of immunity in sows before they are infected with PEDV is one of the important factors to prevent PED.

The pregnant sows acclimated using feces or intestinal contents from piglets with PED may induce mucosal immunity to PEDV. Feedback is expected to induce high-level immunity by a booster effect when combined with vaccination. However, carries risks, including a tremendous increase in viral load of the farm and introduction of pathogens such as porcine reproductive and respiratory syndrome virus, porcine circovirus type 2, rotavirus, porcine parvovirus, porcine enterovirus, *Salmonella* spp., *Lawsonia intracellularis*, *Escherichia coli*, and *Erysipelothrix rhusiopathiae*, etc. via infective materials. PEDV included in infective materials cannot be stored for a long time in general home-use freezers. The use of sufficient virus level is not ensured in PED re-break. Because it is challenging to control feedback and provide stable results completely, Japanese government published the PED prevention manual to guide farmers not to perform feedback unless veterinarians or applicable government agencies are involved [21].

Two vaccines based on the genogroup 2 virus have already been available in the US; however, the efficacy is limited. Crawford et al. reported the potency of the vaccine which developed by Harrisvaccines™ in Iowa in naïve sows [22]. As the results of the study, average litter mortality in the control group was 91%, while average mortality in the vaccinated group was 69%. Even if a vaccine based on the genogroup 2 virus is used, it is hard to confer protective immunity against PEDV.

## Conclusions

This paper discusses the effect of the PED live vaccine currently available in Japan as examined in laboratory testing and field trials. Although the PED live vaccine could not protect sows from the viral infection, it provided some effects of protection on field strains in new genetic groups. Damage from PED has been reported even from vaccinated farms in Japan. An ideal vaccine to overcome these problems would provide sows with mucosal immunity and piglets with more potent lactogenic immunity to protect both of them from the viral infection. Further research would be warranted to develop such an ideal vaccine by more closely understanding the pathogenic mechanism of PED, examining other immunization methods instead of intramuscular injection, and developing an effective antigen to induce the mucosal immune response.

## Additional file

Additional file 1: Table S1. The number of dead piglets by age in weeks at field farms with PED. ^a^Weeks are indicated as follows: 0 = the mortality rate of piglets born in the week of PED outbreak, − 1, − 2 or − 3 = the mortality rate of piglets in 1, 2, or 3 weeks of age at the week of PED outbreak, respectively, 1 or 2 = the mortality rate of piglets born in the next weeks of PED outbreak. (PDF 34 kb)

## Abbreviations

ANOVA: Analysis of variance
CPE: Cytopathic effect
INDEL: Insertions and deletions in the spike gene
MEM: Minimum essential medium
PED: Porcine epidemic diarrhea
PEDV: Porcine epidemic diarrhea virus
RT-qPCR: reverse transcription-quantitative polymerase chain reaction
TCID_50_: 50% tissue culture infective dose
TGE: transmissible gastroenteritis
TPB: Tryptose phosphate broth
