# Supplementary material for: Efficacy of genogroup 1 based porcine epidemic diarrhea live vaccine against genogroup 2 field strain in Japan
Source: Virol J. 2018 Feb 2;15:28. doi: 10.1186/s12985-018-0940-8 (PMC5797392; doi:10.1186/s12985-018-0940-8)
Supplement: Additional file 1: Table S1. — The number of dead piglets by age in weeks at field farms with PED. aWeeks are indicated as follows: 0 = the mortality rate of piglets born in the week of PED outbreak, − 1, − 2 or − 3 = the mortality rate of piglets in 1, 2, or 3 weeks of age at the week of PED outbreak, respectively, 1 or 2 = the mortality rate of piglets born in the next weeks of PED outbreak. (PDF 34 kb) [file 12985_2018_940_MOESM1_ESM.pdf]

Table S1: The number of dead piglets by age in weeks at field farms with PED.

	Weeks[a]					
Farm	-3	-2	-1	0	1	2
Farm A	67/403	104/401	210/396	380/396	112/401	2/410
Farm B	2/498	44/695	193/522	354/370	284/444	134/459
Farm C	2/190	58/200	444/500	583/596	552/610	109/190

[a]Weeks are indicated as follows: 0=the mortality rate of piglets born in the week of PED outbreak, -1, -2 or -3=the mortality rate of piglets in 1, 2, or 3 weeks of age at the week of PED outbreak, respectively, 1 or 2=the mortality rate of piglets born in the next weeks of PED outbreak.
